# Proteomics, modeling, and fluorescence assays delineate cytochrome *b*_5_ residues involved in binding and stimulation of cytochrome P450 17A1 17,20-lyase

**DOI:** 10.1016/j.jbc.2024.105688

**Published:** 2024-01-26

**Authors:** Yasuhiro Tateishi, Stephany N. Webb, Bian Li, Lu Liu, Kristie Lindsey Rose, Micheal Leser, Purvi Patel, F. Peter Guengerich

**Affiliations:** 1 Department of Biochemistry, Vanderbilt University School of Medicine, Nashville, Tennessee, USA; 2 Department of Medicine, Vanderbilt University Medical Center, Nashville, Tennessee, USA; 3 Proteomics Laboratory, Mass Spectrometry Research Center, Vanderbilt University School of Medicine, Nashville, Tennessee, USA

**Keywords:** cytochrome P450, cytochrome *b*_5_, proteomics, fluorescence, protein-protein interactions, steroidogenesis

## Abstract

Cytochrome *b*_5_ (*b*_5_) is known to stimulate some catalytic activities of cytochrome P450 (P450, CYP) enzymes, although mechanisms still need to be defined. The reactions most strongly enhanced by *b*_5_ are the 17,20-lyase reactions of P450 17A1 involved in steroid biosynthesis. We had previously used a fluorescently labeled human *b*_5_ variant (Alexa 488-T70C-*b*_5_) to characterize human P450 17A1-*b*_5_ interactions, but subsequent proteomic analyses indicated that lysines in *b*_5_ were also modified with Alexa 488 maleimide in addition to Cys-70, due to disulfide dimerization of the T70C mutant. A series of *b*_5_ variants were constructed with Cys replacements for the identified lysine residues and labeled with the dye. Fluorescence attenuation and the function of *b*_5_ in the steroid lyase reaction depended on the modified position. Apo-*b*_5_ (devoid of heme group) studies revealed the lack of involvement of the *b*_5_ heme in the fluorescence attenuation. A structural model of *b*_5_ with P450 17A1 was predicted using AlphaFold-Multimer algorithms/Rosetta docking, based upon the individual structures, which predicted several new contacts not previously reported, that is, interactions of *b*_5_ Glu-48:17A1 Arg-347, *b*_5_ Glu-49:17A1 Arg-449, *b*_5_ Asp-65:17A1 Arg-126, *b*_5_ Asp-65:17A1 Arg-125, and *b*_5_ Glu-61:17A1 Lys-91. Fluorescence polarization assays with two modified *b*_5_ variants yielded *K*_d_ values (for *b*_5_-P450 17A1) of 120 to 380 nM, the best estimate of binding affinity. We conclude that both monomeric and dimeric *b*_5_ can bind to P450 17A1 and stimulate activity. Results with the mutants indicate that several Lys residues in *b*_5_ are sensitive to the interaction with P450 17A1, including Lys-88 and Lys-91.

Cytochrome *b*_5_ (*b*_5_, CYB5A) is a small (∼18 kDa) accessory protein involved in several reactions, including fatty acid desaturation. It is involved in several cytochrome P450 (P450, CYP)-catalyzed reactions, including xenobiotic metabolism, fatty acid metabolism, and steroid biosynthesis, either inhibiting or stimulating. In some cases there is evidence that *b*_5_ transfers the second electron (received from NADPH-cytochrome P450 reductase (POR) or NADH-cytochrome *b*_5_ reductase) to the Fe^2+^O_2_ complex of P450 (1, 2), but in many cases *b*_5_ is believed to act as an allosteric partner, modulating the conformation of P450s upon binding (3, 4, 5). However, a definitive mechanism of this function remains unclear in the absence of binary crystal structures.

One of the enzymes whose catalytic activity is strongly enhanced by *b*_5_ is P450 17A1, also known as steroid 17α-hydroxylase/17,20-lyase. The enzyme is localized in steroidogenic tissues (e.g., adrenal glands, testis, and ovaries) and mainly catalyzes 2-step oxidations of steroids, namely 17α-hydroxylation and the subsequent 17,20 C-C bond cleavage (the so called “lyase” reaction), to yield androstenedione and dehydroepiandrostenedione (Fig. 1). Although these two reactions are the major ones involved with progesterone and pregnenolone, some minor pathways (*e.g.*, 16α-hydroxylation) are also known (6). P450 17A1 plays an essential role in producing androgens as well as 17α-hydroxy steroids, which are further converted to mineralocorticoids and glucocorticoids. The enzyme is involved in some human maladies, including breast cancer (7), polycystic ovary syndrome (8, 9), Cushing’s syndrome (10), glioblastoma (11), and particularly prostate cancer (12). Although numerous efforts have been devoted to developing P450 17A1 inhibitors, only one drug, abiraterone (prodrug abiraterone acetate), has been approved for the treatment of castration–resistant prostate cancer. However, this drug is known to have major side effects because of its nonspecific inhibition of other P450 enzymes (13, 14, 15) and nonselectivity between the 17α-hydroxylation and 17,20-lyase reactions of P450 17A1 (Fig. 1) (12, 16). Therefore, the discovery of more selective lyase inhibitors (which would only reduce the production of androgens) is still desired. The *b*_5_-P450 17A1 interaction is of great interest from both clinical and biochemical viewpoints because *b*_5_ has been shown to play an essential role in 17,20-lyase reactions catalyzed by P450 17A1 but has little or no effect on 17α-hydroxylations (17, 18, 19).

**Figure 1:**
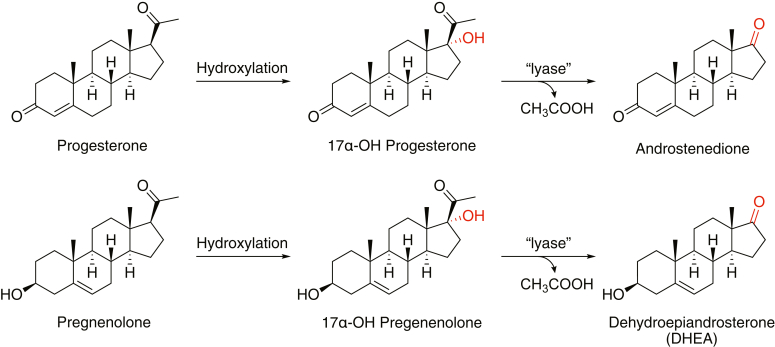
**Major reactions catalyzed by P450 17A1**.

Structures of both human *b*_5_ and P450 17A1 (20) are available, but to our knowledge no crystal structure of a *b*_5_-P450 17A1 protein complex has been reported. Nevertheless, many studies have been published on the functions of this interaction. It is generally accepted that *b*_5_ allosterically stimulates lyase reactions by inducing conformational changes of P450 17A1, not by electron transfer from *b*_5_ to P450 17A1 (4, 21, 22). More than 125 clinical variants of P450 17A1 have been reported (20), and some of these (e.g., R347C, R347H, R347Q, R358Q, P428L, F417C, and E305G) are known to preferentially affect the lyase activity *in vivo* (23, 24, 25). The variants R347H, R347Q, and R358Q are considered to be deficient due to the loss of ability to bind to *b*_5_ (23, 26, 27). Similarly, the Glu-48 and Glu-49 residues of *b*_5_ are critical for the lyase activity (28) and clinical variants are known with serious endocrinological issues (29, 30). The importance of these residues is further supported by experimental data utilizing site-directed mutagenesis, NMR spectroscopy, and chemical crosslinking, indicating that ionic interactions between acidic residues of *b*_5_ and basic residues of P450 17A1 are critical (28, 31, 32).

One of the issues in the field is that there are few useful assays for the analysis of the interactions between *b*_5_ and P450 enzymes. Surface plasmon resonance-based assays have been used with *b*_5_ and P450 17A1, with a reported *K*_d_ value of 440 nM (33). However, this approach has caveats due to the need to immobilize one protein and a major deficiency is “mass transfer,” a term used to describe the diffusion of the ligand from the solution through the matrix to reach the receptor (P450 17A1) (34). NMR spectroscopy has also been used to investigate this interaction qualitatively (35), but this approach has issues in estimating the low *K*_d_ value because high concentrations of the proteins are required. Simonov *et al*. (21) reported FRET-based assays that used fluorescent protein-fused P450 17A1 and *b*_5_ to successfully demonstrate the interaction within cells. We have used a fluorescently labeled *b*_5_ T70C variant, observing the fluorescence attenuation by titrating with P450 enzymes (19, 36).

To better understand *b*_5_-P450 17A1 interactions, we further investigated fluorescence-based protein-protein interaction assays. Site-directed mutagenesis was conducted to construct a series of Lys-to-Cys *b*_5_ variants for selective labeling, based on the lysine residues modified with Alexa Fluor 488 maleimide. A structure for *b*_5_-P450 17A1 complex was modeled via AlphaFold-Multimer (AFM) protein complex structure prediction (37) and Rosetta protein-protein docking (38). The modeled structure agrees well with existing knowledge about *b*_5_-P450 17A1 interaction and also suggests potential new interactions for further experimental investigation. The fluorescence attenuation of Alexa 488-conjugated *b*_5_ mutants (by P450 17A1) varied depending on the labeled position. A fluorescence polarization assay was also developed to study the binding, and the *K*_d_ value of the binding was estimated in the sub-μM range (130 nM under these conditions).

## Results

### Preparation of Alexa 488-labeled WT human b_5_ and titration with P450 17A1

**Figure 2:**
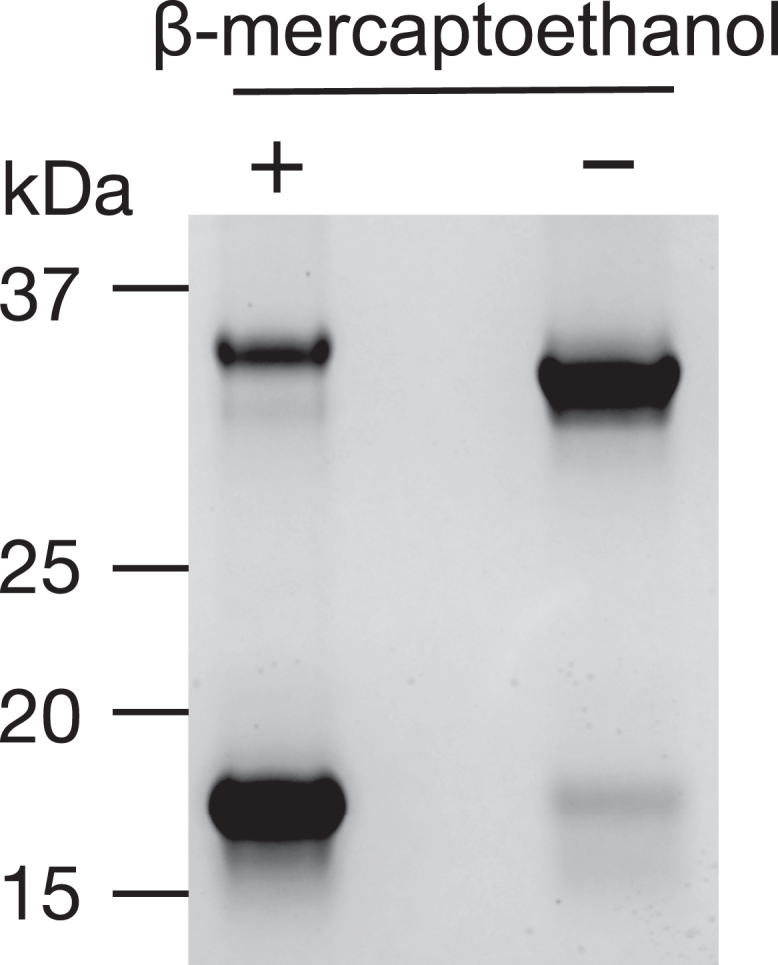
**SDS-PAGE of previously construct of Alexa 488-T70C-*b***_**5**_ (19). Proteins were separated on a 10% Bis-tris polyacrylamide gel with MES running buffer. Alexa 488-T70C-*b*_5_ (200 pmol) was loaded either with (*left lane*) or without (*right lane*) pretreatment with β-mercaptoethanol (10%, v/v). The gel was imaged by total protein staining by SimplyBlue Safe Stain (Thermo Fisher Scientific). The monomer migrated at 17 kDa and the dimer at 34 kDa, with some difference in mobility of the dimer in the sample containing β-mercaptoethanol. MES, 2-[*N*-morpholino]ethanesulfonic acid.

In previous work, we labeled a human *b*_5_ mutant (T70C) with a green fluorophore (Alexa Fluor 488 C5 maleimide) and used it to characterize the interaction between *b*_5_ and human P450s (19, 36). In the course of further studies with Alexa 488-labeled T70C *b*_5_ (Alexa 488-T70C-*b*_5_), we found that this protein exists mainly as a dimer that was disrupted by thiols (e.g., high concentrations of DTT), implying that the thiol in the cysteine residue was forming a disulfide bond instead of reacting with the maleimide group of Alexa 488 dye (Fig. 2). The requirement for a high concentration of DTT (10 mM, Fig. S1) was surprising in light of our finding of near-maximal stimulation of the laurate ω-hydroxylation activity of P450 4A11 by 50 μM DTT or tris(2-carboxyethyl)phosphine (39). We also found that tris(2-carboxyethyl)phosphine was not effective in reducing the disulfide linkage of the *b*_5_ dimer (Fig. S1). (In a previous study with rat *b*_5_ and bacterial P450 101A1, Stayton *et al*. (40) had used 10 mM DTT to reduce the corresponding (rat *b*_5_) T65C mutant prior to modification with another reagent but did not comment on the need for use of a high concentration to prevent dimerization.)

**Figure 3:**
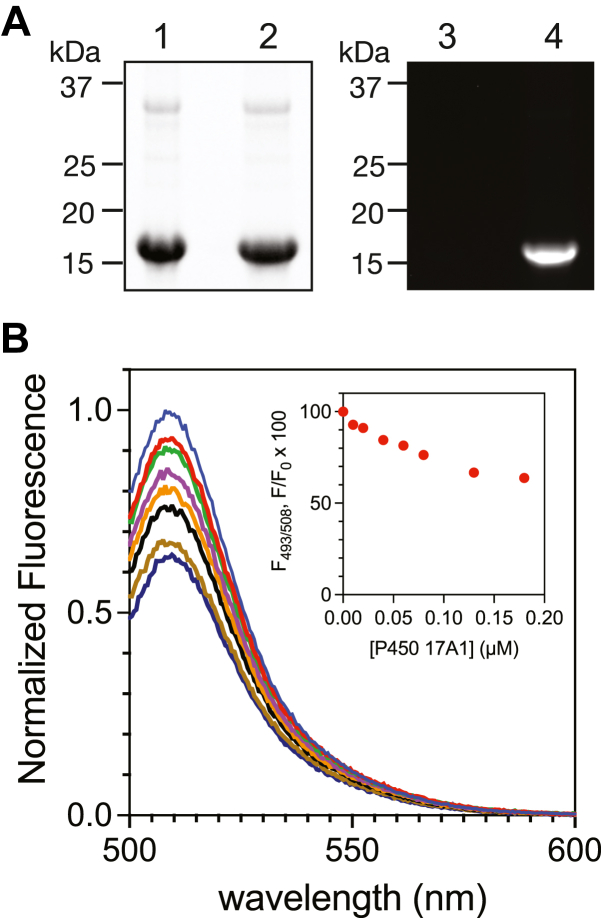
**Construction of Alexa 488-WT-*b***_**5**_**and fluorescence titration with P450 17A1.***A*, SDS-PAGE of nonlabeled (lanes 1, 3) or Alexa 488-labeled WT *b*_5_ (lanes 2 and 4). The gel was imaged by total protein staining by SimplyBlue Safe Stain (Thermo Fisher Scientific) (lanes 1 and 2) or excited with 493 nm light (lanes 3 and 4); *B*, a solution of Alexa 488-WT *b*_5_ (50 nM) was excited at 493 nm in a spectroflourimeter and the attenuated emission spectra (500–600 nm) were recorded following addition of increasing concentrations of P450 17A1. The emission spectra were normalized to the initial (maximum) fluorescence intensity. The inset shows the normalized fluorescence data points at the emission wavelength maximum (508 nm) in fluorescence titrations.

Based on this observation, monomeric Alexa 488-labeled WT *b*_5_ (Alexa 488-WT-*b*_5_, devoid of Cys residues) was prepared by reaction with the fluorophore overnight at room temperature, using the same conditions as previously mentioned (19) (Fig. 3*A*). The product was desalted and titrated with P450 17A1 in 1 mM potassium phosphate buffer. The fluorescence intensity was attenuated by the addition of P450 17A1 (Fig. 3*B*), as observed previously in the case of Alexa 488-T70C-*b*_5_ (19). Only minimal inner-filter effects (∼3%) were observed within the range of P450 17A1 concentrations we examined (Fig. S2). The addition of WT *b*_5_ eliminated the attenuation of fluorescence intensity, providing evidence that the observed fluorescence attenuation was due to the binding of the modified *b*_5_ to P450 17A1 (Fig. S3, *A* and *B*). Titration in buffer with higher ionic strength (100 mM potassium phosphate) resulted in less attenuation of fluorescence (Fig. S3, *C* and *D*), consistent with previous reports that the interaction between *b*_5_ and P450 17A1 involves ionic interactions (31, 32).

### Mechanistic study of fluorescence attenuation using apo-b_5_

**Figure 4:**
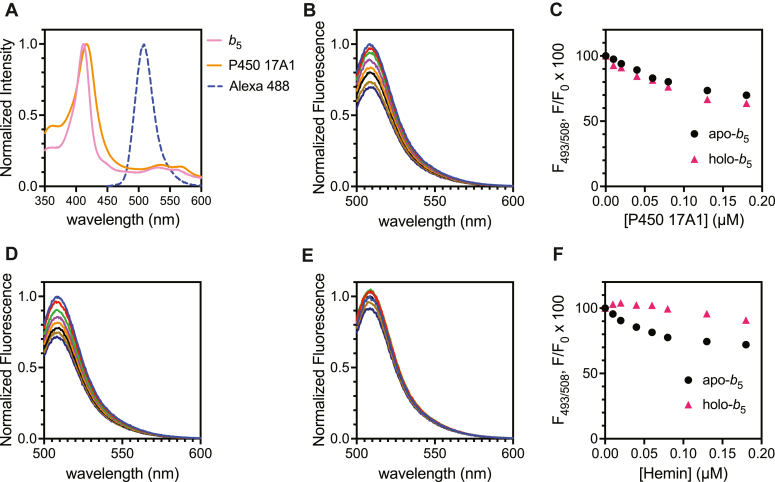
**Effect of heme in *b***_**5**_**on fluorescence titration.***A*, normalized absorbance spectra (*solid line*) of *b*_5_ (*pink*) or P450 17A1 (*orange*) and fluorescence (emission) spectra of Alexa 488 (*stippled line*, *blue*); *B*, representative normalized fluorescence spectra of Alexa 488-apo-*b*_5_ (50 nM) with increasing concentrations of P450 17A1; *C*, F/F_0_ plot of the normalized fluorescence when titrated with P450 17A1. (The data with holo-*b*_5_ (Alexa 488-WT *b*_5_) were the same as in the experiment of Fig. 3*B*); *D* and *E*, representative normalized fluorescence spectra of Alexa 488-apo-*b*_5_ (*D*) and Alexa 488-WT *b*_5_ (*E*) with increasing concentrations of hemin; *F*, F/F_0_ plot of the normalized fluorescence when titrated with hemin.

The absorbance spectra of P450 17A1 and *b*_5_ showed considerable overlap with the absorbance (excitation) spectrum of the Alexa-488 fluorophore (Fig. 4*A*), suggesting the possibility that the heme in one or both enzymes is related to the observed fluorescence attenuation. Accordingly, the apo form of *b*_5_ (apo-*b*_5_) was prepared by treating WT *b*_5_ (holo form) with acid in acetone (3, 41) to remove the heme. Apo-*b*_5_ was modified with Alexa 488 to prepare Alexa 488-apo-*b*_5_ (using the same procedure used to prepare Alexa 488-WT-*b*_5_) and then titrated with hemin or P450 17A1. The fluorescence of both Alexa-modified apo- and holo-*b*_5_ was attenuated to a similar extent (Figs. 3*B*, and 4, *B* and *C*). When titrated with hemin, the fluorescence of Alexa 488-apo-*b*_5_ was attenuated although Alexa 488-WT *b*_5_ (holo-form) showed little quenching upon titration (Fig. 4, *D*–*F*). No change in the fluorescence spectra of free Alexa 488 C5 maleimide dye was observed following the addition of either hemin or P450 17A1 (Fig. S4). Collectively these results are interpreted to mean that the observed attenuation of Alexa 488-*b*_5_ fluorescence was not due to the *b*_5_ heme prosthetic group, but the attenuation of fluorescence can be due to either the protein P450 17A1 or possibly its heme group.

### Proteomic analysis of sites of Alexa 488 labeling in modified b_5_

In light of the results demonstrating the covalent binding of Alexa 488 to *b*_*5*_ dimer (Fig. 2), in which the only Cys residue in the T70C mutant *b*_5_ was tied up in a disulfide linkage, we hypothesized that lysine residues in WT *b*_5_ reacted with the maleimide group of Alexa 488, based on established literature precedent (42). Accordingly, the fluorescence attenuation in titrations with P450 17A1 would be linked with alterations in the environments of these residues. To determine which amino acids had reacted with the Alexa 488 fluorophore, proteomic analysis of Alexa 488-WT-*b*_5_ was conducted. Following SDS gel electrophoresis, *b*_5_ was digested with trypsin and the resulting peptides were analyzed by high-resolution mass spectrometry (Table S1). The data indicated that six of the eight lysines (Lys-10, Lys-19, Lys-24, Lys-33, Lys-39, and Lys-77), as well as the *N-*terminal amino group in WT *b*_5_, were labeled with the Alexa fluorophore (tandem mass spectra of each modified peptide are shown in Fig. S5, and a list of detected peptides is shown in Table S2).

### Computational modeling of the structure of b_5_-P450 17A1 complex

**Figure 5:**
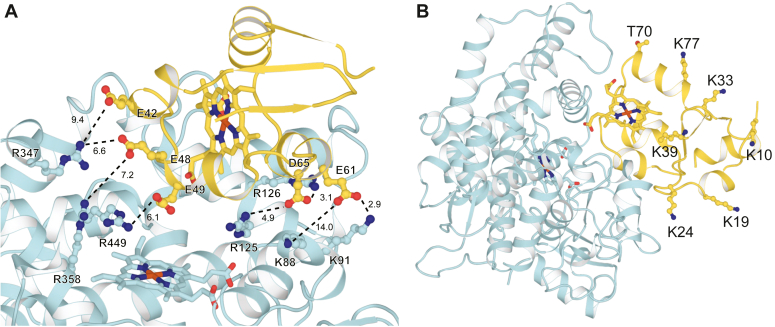
**AlphaFold-Multimer-derived models of *b***_**5**_**:P450 17A1 binary complex.***A*, the two negatively charged patches in *b*_5_ (*yellow*), *i.e.* E42, E48, E49 and E61, D65 are in close proximity with positively charged patches in P450 17A1 (*cyan*), *i.e.* R347, R358, R449 and K91, R125, R126. Interestingly, the P450 17A1 residue K88, which was reported by Peng *et al*. (32) to interact with E61 in *b*_5_, is relatively far away from E61 in our model. Given the long sidechain of K88, it is likely that K88 can interact with E61 after adopting a different side-chain rotamer. Numbers next to *dashed lines* represents the distances (in Å) between the corresponding pair of atoms. *B*, locations of *b*_5_ residues K10, K19, K24, K33, K39, T70, and K77. Their distances to the *b*_5_-heme and P450 17A1-heme are tabulated in Table 1.

A 3-dimensional structural model of P450 17A1 bound to *b*_5_ was built by combining deep-learning based multimeric protein structure prediction and biophysics-based modeling and docking (see Experimental Procedures). Briefly, AFM algorithms (37) were used to generate a starting model of a *b*_5_-P450 17A1 complex. This model was then energy-minimized and used to perform protein-protein docking to further sample the binding poses between *b*_5_ and P450 17A1 using the Rosetta modeling suite (38). The model revealed that Arg-347 and Arg-358 in P450 17A1 are close to Glu-48 and Glu-49 in *b*_5_, as reported in chemical crosslinking studies by Peng *et al*. (32) and other lines of investigation (23, 24, 28, 31), providing confidence about the results of the final model (Fig. 5*A*, Tables 1, and 2). Among the Alexa 488-labeled amino residues that we identified in *b*_5_ (see above), the *N*-terminus and Lys-10 are located relatively far away from the heme prosthetic groups in P450 17A1 and from the putative site of interaction (Fig. 5*B*). Thus, we focused on the other five positions, that is, Lys-19, Lys-24, Lys-33, Lys-39, and Lys-77 in the following investigations.

**Table 1:** Distances (in Å) between heteroatoms in side chains of some *b*_5_ residues and the P450 17A1 heme iron atom

Residue	Atom	Distance, Å
Lys-10	Nζ	40.1
Lys-19	Nζ	36.9
Lys-24	Nζ	29.8
Lys-33	Nζ	36.2
Lys-39	Nζ	32.6
Thr-70	O	28.9
Lys-77	Nζ	33.3

**Table 2:** Distances (in Å) between *b*_5_ oxygen and P450 17A1 nitrogen atoms of interacting residues identified by modeling (Fig. 5*A*)

*b*_5_ residue	P450 17A1 residue	Distance, Å	Previous report
Glu-42	Arg-347	9.4	(32)
Glu-48	Arg-347	6.6	
Glu-48	Arg-358	7.2	(32)
Glu-49	Arg-449	6.1	
Asp-65	Arg-126	3.1	
Asp-65	Arg-125	4.9	
Glu-61	Lys-88	14.0	(32)
Glu-61	Lys-91	2.9	

### Preparation of Alexa 488-labeled human b_5_ mutants and titration with P450 17A1

**Figure 6:**
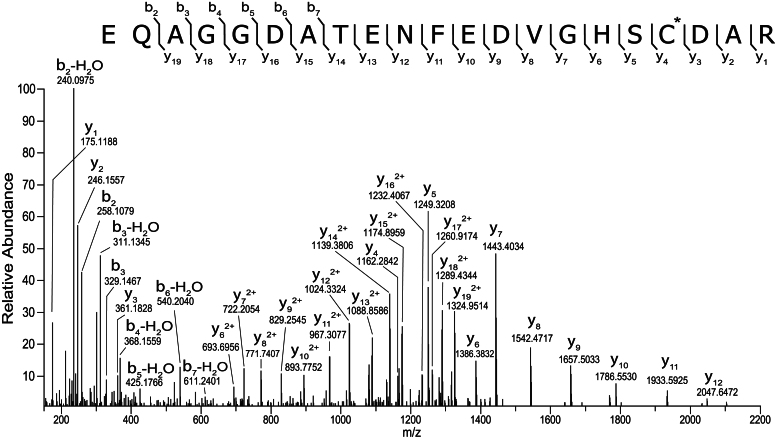
**Tandem mass spectrum of peptide, EQAGGDATENFEDVGHSCDAR, modified at C70 with the Alexa488.** The [M+3H]^3+^ precursor ion was selected for fragmentation, and the observed b- and y-type product ions are assigned to their corresponding *m/z* peaks in the mass spectrum. The amino acid sequence is provided above the annotated spectrum with the position of the Alexa 488 fluorophore (+698.0989) modification denoted by the asterisk (∗), and sites of amide bond fragmentation are indicated with interresidue brackets.

To modify single amino acid residues in *b*_5_, a series of mutant *b*_5_ proteins with cysteine substituted for individual lysines (K19C, K24C, K33C, K39C, and K77C) was constructed (Table S3). Gel electrophoresis in the absence and presence of β-mercaptoethanol showed that the T70C and K24C mutants existed largely as disulfide dimers. All of the mutant *b*_5_ enzymes were treated with 10 mM DTT (see above) prior to labeling with Alexa 488 C5 maleimide to prevent formation of disulfide-linked dimers and then reacted with a 5-fold molecular excess of Alexa 488 C5 maleimide at room temperature for 2 h, conditions designed for selective modification of the single Cys in each protein. Pretreatment with the reducing reagent DTT yielded essentially quantitative reduction of any linked dimers (as judged by gel analysis), which were then reacted with a 5-fold molecular excess of Alexa 488 C5 maleimide at room temperature. The labeling efficiency was nearly quantitative, based on the calculations with the absorbance spectra, and the presence of only a single labeled *b*_5_ peptide at the mutated Cys was confirmed by the proteomic analysis (Fig. 6, Tables S1, and S2, showing T70C *b*_5_ data).

**Figure 7:**
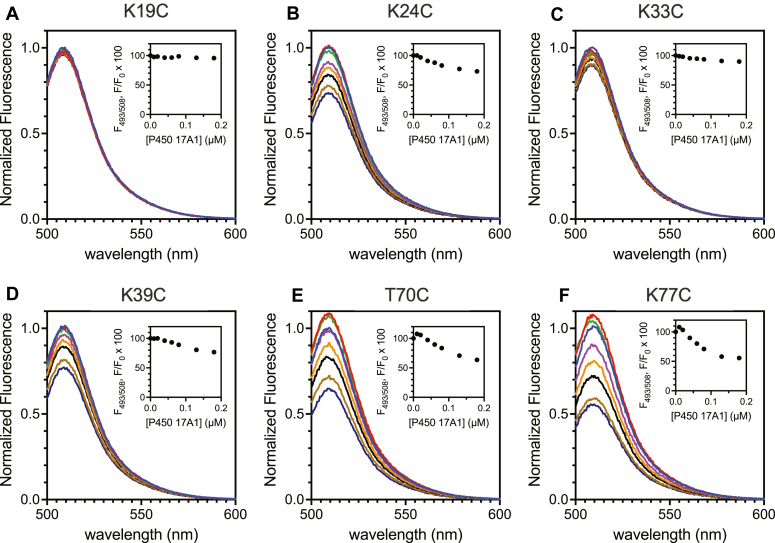
**Fluorescence titration of Alexa 488-mutant *b***_**5**_**s with P450 17A1.** Titrations were performed in 1 mM potassium phosphate buffer (pH 7.4) with Alexa 488-labeled human *b*_5_ variants (50 nM) and P450 17A1 (0, 0.01, 0.02, 0.04, 0.06, 0.08, 0.13, and 0.18 μM). The emission spectra were normalized to maximum fluorescence intensity of each Alexa 488-labeled *b*_5_ mutant. The insets show the normalized fluorescence data points at the emission wavelength maximum (508 nm) in fluorescence titrations. *A*, Alexa 488-K19C-*b*_5_; *B*, Alexa 488-K24C-*b*_5_; *C*, Alexa 488-K33C-*b*_5_; *D*, Alexa 488-K39C-*b*_5_; *E*, Alexa 488-T70C-*b*_5_; and *F*, Alexa 488-K77C-*b*_5_.

Titration of all the Alexa 488-modified mutant *b*_5_ proteins and P450 17A1 showed some loss of fluorescence intensity, as observed in Alexa 488-WT *b*_5_ (Fig. 3*B*), but the character of the saturation curves depended on the site of labeling (Fig. 7). Alexa 488-WT, -T70C, and -K77C-*b*_5_ showed the greatest attenuation (Figs. 3*B*, and 7, *E* and *F*), Alexa 488-K24C and -K39C-*b*_5_ showed moderate changes (Fig. 7*B*, 7*D*), and Alexa 488-K19C and -K33C-*b*_5_ had little change upon titration (Fig. 7, *A* and *C*). Interestingly, Alexa 488-T70C and -K77C-*b*_5_ showed slightly enhanced fluorescence intensity with the addition of a very low concentration of P450 17A1 (∼8% increase), followed by attenuation up to and beyond a stoichiometric concentration (Fig. 7, *E* and *F*). The attenuation of fluorescence intensity caused by the binding of P450 17A1 to these modified *b*_5_ proteins could also be eliminated by the addition of unlabeled WT *b*_5_ (Fig. S6), indicating that the observed fluorescence attenuation of labeled *b*_5_ was due to the binding to P450 17A1.

The K19C mutant, which did not show very much fluorescence attenuation after labeling (Fig. 7*A*), was examined further. SDS-PAGE indicted that the *M*_r_ of this protein was ∼3 kDa lower than that of WT *b*_5_ or the other mutants (Fig. S7), and accordingly it was not used in further analyses (the basis of the lower *M*_r_ was not analyzed).

### Catalytic activity of P450 17A1 enhanced by unmodified or modified b_5_ proteins

**Figure 8:**
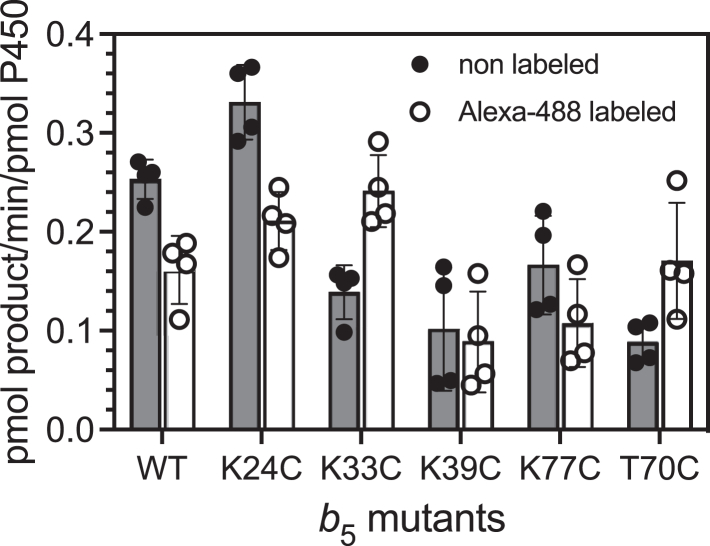
**Stimulation of the P450 17A1-catalyzed 17α-OH pregnenolone 17,20-lyase reaction by unlabeled or Alexa 488-labeled WT *b***_**5**_**and variants.** P450 17A1 (25 pmol) was reconstituted with POR (50 pmol) and *b*_5_ (250 pmol) in the presence of 2 μM 17α-OH pregnenolone. The results are represented as means ± SD of quadruplicate samples obtained from two replicate experiments. POR, NADPH-cytochrome P450 reductase.

It is well-established that human and other P450 17A1 enzymes require *b*_5_ for the steroid 17,20-lyase reaction (17, 19, 43, 44). To examine if the Alexa 488 labeling of *b*_5_ causes the loss of P450 17A1 activity, the lyase activity was measured using our previously reported LC-MS procedure (16, 45) (Fig. 8). With most of the mutant *b*_5_ proteins, except for K24C, lyase rates were only somewhat lower compared to WT *b*_5_. Lyase activity was below the limit of detection without the addition of *b*_5_, as described previously (16, 19, 45).

We measured the rates of electron transfer from NADPH to POR to WT *b*_5_ and each of the *b*_5_ mutants (Fig. S8 and Tables S4). Reduction of WT *b*_5_ was rapid, as expected (46). Rates of electron transfer were compromised for all the mutants, and these rates did not bear a relationship to the stimulation of lyase activity. This result is not surprising, in the light of evidence against a requirement for electron transfer to or from *b*_5_ in stimulation (2, 4, 22, 47). However, even the lowest rates (*e.g.*, with K70C-*b*_5_, Table S4) were faster than the lyase reaction, so they may not be incompatible with a role of ferrous *b*_5_. The roles of the individual lysine residues (and Thr-70) in electron transfer and (most likely) in binding of POR and *b*_5_ are unknown and, to our knowledge, have not been examined by others.

The Alexa 488 probe did not impair the function of *b*_5_ very much in most cases, and with K33C-*b*_5_ and T70C-*b*_5_, the enzyme activity was somewhat enhanced by fluorescent labeling (note that the Eyring-Polanyi equation equates a 2-fold rate change with <0.5 kcal mol^-1^ change in free energy (48), thus small differences only reflect very modest changes in the free energy of binding parameters). We conclude that the fluorescence changes observed with labeled *b*_5_ proteins are indicative of productive interactions with P450 17A1.

### Fluorescence polarization assays of binding of b_5_ to P450 17A1

**Figure 9:**
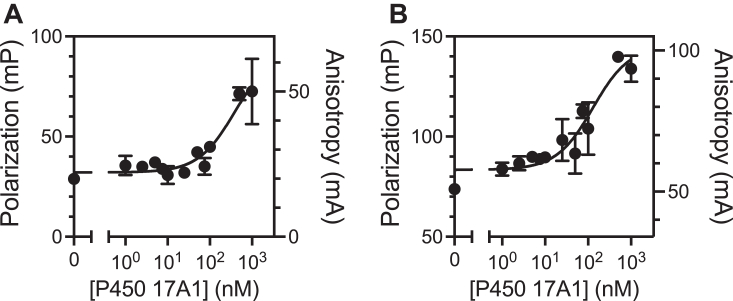
**Fluorescence polarization of Alexa 488-labeled T70C and K77C *b***_**5**_**with P450 17A1.** Increasing concentrations of P450 17A1 were added to (*A*) 10 nM Alexa 488-T70C-*b*_5_ or (*B*) Alexa 488-K77C-*b*_5_ in a 384-well plate (final volume 50 μl). Each value represents the mean ± range of duplicate assays. The estimated *K*_d_ values were 379 and 125 nM, respectively.

Fluorescence polarization is a widely accepted method for the study of protein-protein, protein-peptide, and protein-nucleic acid interactions (49). The photochemical properties of Alexa 488 dye are known to be applicable to fluorescence polarization methods, and accordingly Alexa 488 labeled *b*_5_ was used to develop a fluorescence polarization assay with P450 17A1. The Alexa 488-T70C and -K77C-*b*_5_ proteins showed the most fluorescence attenuation in the P450 17A1 titrations (Fig. 7). A preliminary study suggested that these two modified proteins showed a greater dynamic range compared with other modified *b*_5_ mutants (data not presented), and the calculated *K*_d_ values were 379 and 125 nM, respectively (Fig. 9, *A* and *B* and Table 3). Increasing the ionic strength of the binding buffer was unfavorable for detection of fluorescence polarization (Fig. S9), consistent with previous reports on *b*_5_:P450 17A1 interactions (31, 32). Alexa 488-K77C-*b*_5_ also showed fluorescence polarization with other P450 enzymes in a concentration-dependent manner (Fig. S10), suggesting the general applicability of the fluorescence polarization-based assay for the study of *b*_5_-P450 interactions.

**Table 3:** Binding affinities of Alexa-488 labeled mutant *b*_5_ proteins to P450 17A1 or 17A1 peptide estimated from fluorescence polarization measurements (Figs. 9 and 10)

Alexa 488-mutatnt *b*_5_	P450 17A1	*R*^2^	P450 17A1 peptide	*R*^2^
	*K*_d_, nM (mean ± SEM)		*K*_d_, μM (mean ± SEM)	
T70C	379 ± 240	0.815	113 ± 12	0.984
K77C	125 ± 56	0.827	52 ± 9	0.957

**Figure 10:**
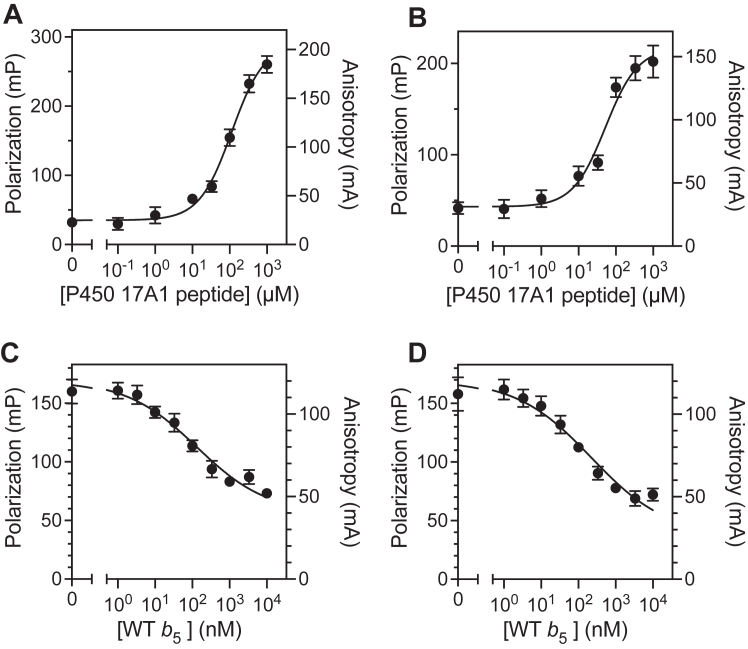
**Fluorescence polarization of Alexa 488-labeled T70C and K77C *b***_**5**_**with P450 17A1 peptide.***A* and *B*, Alexa 488-*b*_5_ T70C (*A*) or K77C (*B*) variants (10 nM) were incubated with various concentrations of the P450 17A1 peptide. Each point represents a mean ± SD of quadruplicate data. The estimated *K*_d_ values were 113 μM (T70C, *A*) and 52 μM (K77C, *B*); *C* and *D*, Alexa 488-*b*_5_ T70C (*C*) or K77C (*D*) variants (10 nM) and P450 17A1 peptide (100 μM) complex was incubated with various concentrations of WT *b*_5_ (nonlabeled). Each point represents a mean ± SD of triplicate data. The estimated IC_50_ value of WT *b*_5_ was 129 ± 41 nM (for T70C) and 223 ± 74 nM (for K77C).

In fluorescence polarization work, it is common to label low *M*_r_ peptides with fluorescent probes. In our previous study, a peptide consisting of the putative *b*_5_ binding site of P450 17A1 (residues 348–358, “P450 17A1 peptide”) showed weak inhibitory activity against the lyase reaction in a reconstituted system (19). This P450 17A1 peptide was added to an Alexa 488-labeled *b*_5_:P450 17A1 complex, with the expectation that the peptide would disrupt the polarization in a concentration-dependent manner. However, the peptide increased the polarization instead of decreasing it, indicating that the P450 17A1 peptide was binding to *b*_5_ (results not shown). Accordingly, fluorescence polarization assays with the P450 17A1 peptide were conducted using Alexa 488-T70C and -K77C-*b*_5_ (Fig. 10, *A* and *B*). The P450 17A1 peptide produced fluorescence polarization (Fig. 10, *A* and *B*; *K*_d_ values 113 and 52 μM, respectively), although the affinity was weak. This binding was disrupted by the addition of nonlabeled WT *b*_5_ (Fig. 10, *C* and *D*). On the basis of these results, we prepared an Alexa 488-labeled P450 17A1 peptide, which had the modification at either the *N* or *C* terminus. However, no fluorescence polarization was observed when the Alexa 488-labeled P450 17A1 peptide was incubated with concentrations of WT *b*_5_ up to 10 μM, indicating that these modified peptides did not bind to *b*_5_ (Fig. S11), at least not generating a signal.

## Discussion

The 17,20-lyase reactions catalyzed by P450 17A1 (Fig. 1) require *b*_5_, and this complex could be an attractive drug target for the treatment of some human diseases, for example, prostate cancer. We recently developed a fluorescence-based binding assay to visualize this protein-protein interaction (19). In that report, we utilized site-directed mutagenesis to construct a human *b*_5_ T70C mutant and reacted it with an Alexa 488 fluorophore with a maleimide linker (19). However, we subsequently found that this mutant exists as a dimer and was not tagged with the fluorophore site specifically (Fig. 2). WT *b*_5_ (with no cysteine) could be labeled with the maleimide-containing dye under the same conditions (Fig. 3*A*). The labeled WT *b*_5_ also showed fluorescence quenching when titrated with P450 17A1, as previously observed in the labeled T70C mutant (Fig. 3*B*). Accordingly, we cannot make more conclusions about the details of the site of interaction based on that work (19), in that we were uncertain of the position of the fluorophore (Fig. S5 and Table S2).

Our docking computational model *b*_5_-P450 17A1 structure (Fig. 5) is consistent with some of the interactions identified by chemical cross-linking (32) and by numerous site-directed mutagenesis studies (23, 24, 28, 31). In addition to the interactions reported by Peng *et al*. (32), the model (Fig. 5*A*) also identified five more (Table 2). Functional evidence for the importance of these additional interactions has not been confirmed by site-directed mutagenesis or the observation of clinical variants. Arg-449 was implicated in *b*_5_ interaction by Lee-Robichaud *et al*. (24). The apparent multiplicity of interactions might render further site-directed mutagenesis experiments difficult in light of the multiple contributions of individual residues.

It is known that some heme proteins cause fluorescence quenching when tagged with a green fluorophore, which is suggested to be due to the energy transfer from fluorophores to heme (50, 51). In the case of *b*_5_, an enhanced green fluorescent protein-*b*_5_ fusion protein was constructed, and FRET between enhanced green fluorescent protein and heme was reported (52, 53). We investigated this possibility in our case by utilizing apo-*b*_5_ (Fig. 4), which has previously been shown to be effective in stimulating the lyase activity of P450 17A1 (4, 22). Constructing structurally intact apo-P450 is not known to be possible in the case of the mammalian P450s, to our knowledge. The attenuation of the Alexa 488 dye could also be possible via photoinduced electron transfer by interacting with some amino acids (tryptophan and tyrosine), as presented elsewhere (54, 55). However, the environmental changes around Alexa 488 fluorophore do not solely induce fluorescence quenching, in that emission from Alexa 488-tagged adrenodoxin was shown to increase by binding to P450 27C1, which is also a heme protein (56), although Alexa-adrenodoxin fluorescence was attenuated upon binding to another P450 enzyme, 11A1 (57). In fact, a slight increase was observed in Alexa 488-T70C and Alexa 488-K77C-*b*_5_ when mixed with low concentrations of P450 17A1 (Fig. 7, *E* and *F*), indicating the possible contribution of multiple factors for the observed fluorescence attenuation in our experimental systems.

Cysteine-selective labeling was accomplished by pretreating *b*_5_ variants with 10 mM DTT and limited incubation time (Fig. 6 and Table S2). All of the variants except K19C were shown to bind to P450 17A1 and enhance its lyase activity (Figs. 7, 8, and S6). In one of our previous studies, Alexa 488-T70C *b*_5_ (nonspecifically labeled) did not show any apparent binding to P450 2A6 (36) (as reported before on the basis of NMR studies by Bart and Scott (35)) but stimulation of P450 2A6 by *b*_5_ is known (58, 59). Likewise, some inconsistency between binding (of Alexa 488-T70C *b*_5_) and stimulation (by *b*_5_ itself) was observed in the cases of P450s 1A2, 2D6, and 2S1 (36). It may be possible that the presence of the fluorophore prevented labeled *b*_5_ from binding to P450s in these cases, but these examples describe the difficulty of making simple correlations between fluorescence quenching and stimulation of activity. Considering the complexity with the singly labeled Lys-to-Cys *b*_5_ mutants, we did not estimate the binding affinity using the fluorescence attenuation measurements in the present work.

The fluorescence attenuation patterns of the Alexa 488-labeled *b*_5_ mutants are generally consistent with the model we developed (Fig. 5*B*). Lys-24, -39, and −77 and Thr-70 are in positions where the addition of the large fluorophore leads to fluorescence changes upon binding P450 17A1. Lys-19 and Lys-33 are not close (Fig. 5*B*). None of the Lys residues (or Thr-70) appear to be critical in binding of *b*_5_ to P450 17A1, in that the mutants all stimulated 17,20-lyase activity (Fig. 8). The results are consistent with the overall view that the interacting charges on *b*_5_ are all anionic and the charges on P450 17A1 are cationic, although the contribution of other binding forces is possible. The addition of a large fluorophore (Alexa 488) near the binding interface is apparently not enough to eliminate binding, in that stimulation of P450 17A1 17,20-lyase activity was not obliterated.

Another useful fluorescence-based technique to detect protein-protein interactions is fluorescence polarization or fluorescence anisotropy (60). This technology has been used to study protein-protein interactions regarding P450-P450 and P450-POR interactions (61) but to the best to our knowledge, no fluorescence polarization-based assay has been reported for *b*_5_-P450 17A1 interaction. Polarization was observed for *b*_5_ binding to P450 17A1, with *K*_d_ values in the range of 100 to 400 nM (Fig. 9 and Table 1). Fluorescence polarization was also applicable to other P450 enzymes, especially P450 3A4 (Fig. S8), known to be one of the most *b*_5_-stimulated hepatic P450 enzymes (58). One interesting finding is that polarization was also observed by adding the P450 17A1 peptide, R_347_NRLLLLEATIR_358_, which contains the putative binding site of P450 17A1 including Arg-347 and Arg-358 (Fig. 10, *A* and *B*) (numbering based on the amino acid sequence of P450 17A1). This complex was disrupted by adding (nonlabeled) *b*_5_, showing IC_50_ values of 100 to 200 nM, which are close to the binding affinities of Alexa 488-labeled *b*_5_ variants (Fig. 10, *C* and *D*). Estimated *K*_d_ values for the peptide are ∼10^3^-fold higher compared with those of the P450 17A1 protein, indicating the weak interaction as observed with its low inhibitory activity against the lyase reaction (19). Besides Arg-347 and Arg-358, Arg-449 and Lys-88 of P450 17A1 are also considered to be critical basic residues for the *b*_5_-P450 17A1 interaction (24, 31, 32). Our model also shows roles of Lys-91, Arg-125, and Arg-126 (Fig. 5*A* and Table 2). The peptide we used lacked these residues, which could be a potential reason why it showed weak binding. Although interactions with *b*_5_ amino acid residues have not been identified, the P450 17A1 clinical variants P428L, F417C, and E305G are known to have decreased lyase activity (62, 63, 64), suggesting the potential importance of these amino acids, which are also lacking in our peptide. Another potential issue is the presence of the Alexa 488 fluorophore which has a net negative charge. This could be more problematic for Alexa 488-labeled peptides because (intramolecular) ionic interactions between the negative charge on the fluorophore and the positive charge on the amino group of arginine in the peptide might prevent the peptide from binding to *b*_5_. Accordingly, further optimization is necessary to develop a peptide-based fluorescence polarization assay.

We have focused on the roles of positively charged P450 17A1 residues and negatively charged residues of *b*_5_ in the interaction of the two proteins (*e.g.*, Fig. 5), and the dependence of binding on ionic strength (Fig. S9) argues for a major effect of this type. However, other interactions may contribute as well. It is of interest that we reported that the (clinically observed) *b*_5_ mutant E305G did not stimulate lyase activity (as already known) or bind well to Alexa-tagged T70C-*b*_5_ in our earlier work (although the site of Alexa labeling was not defined, as we now know). As pointed out earlier, the P450 17A1 clinical variants P428L and F417C show decrease lyase activity as well.

In summary, we have developed several fluorescence-based methodologies to analyze *b*_5_-P450 17A1 protein-protein interactions. Our previously constructed Alexa 488-T70C-*b*_5_ protein contained multiple fluorophores bound at lysines throughout the protein (19), which we have now corrected to have single labeling at sites of mutated cysteines. Other Alexa 488-modified Lys-to-Cys variants were prepared to study the fluorescence quenching but, considering the possible mechanism of this phenomenon, it is still challenging to interpret the active binding and stimulation of lyase activity by only using fluorescence spectra. We report an alternate fluorescence polarization assay utilizing the constructed fluorescently labeled *b*_5_ variants, which provided what is probably a more accurate sub-μM binding affinity to the P450 17A1 protein (Fig. 9 and Table 3). Although the assays with peptides need to be further developed, this approach can provide opportunities to study the *b*_5_-P450 17A1 interaction. Finally, some modern algorithms have recently been developed not only to predict protein structures but also binding interactions. We have used some of these to further refine the current view of *b*_5_:P450 17A1 interactions (Fig. 5, *A* and *B*), including five new putative ionic (or possibly hydrogen) bonding interactions (Table 2).

## Experimental procedures

### Chemicals and peptides

Alexa Fluor 488 C5 maleimide was purchased from Thermo Fisher Scientific. 17α-OH pregnenolone, dansylhydrazine, and L-α-dilauroyl-*sn*-glycero-3-phosphocholine were purchased from Millipore-Sigma–Aldrich. All other reagents were of analytical grade.

The P450 17A1 peptide (purchased from New England Peptides) was used in our previous report (19). Alexa 488-labeled P450 17A1 peptides were purchased from Thermo Fisher Scientific. The Alexa 488 fluorophore was conjugated to each peptide *via N*-hydroxysuccinimide (for N-terminal modification) or maleimide (for C-terminal modification) linker. The sequence of each peptide and the Alexa 488-modified positions are shown below:

P450 17A1 peptide: R_347_NRLLLLEATIR_358_

N-terminal modified P450 17A1 peptide: [Alexa 488]-ISDRNR_347_LLLLEATIR_358_G

C-terminal modified P450 17A1 peptide: GR_347_LLLLEATIR_358_EVLC-[Alexa 488]

### Enzymes

Recombinant rat POR and human P450 17A1 (with a (His)_6_ tag on the C terminus) were expressed in *Escherichia coli* and purified as described previously (65). The expression plasmids for human *b*_5_ variants were constructed using an Agilent QuikChange II Site-Directed Mutagenesis Kit according to the manufacturer’s instructions. Primer pairs for generating each mutant are shown in Table S3. Expression (*E. coli*) and purification procedure of human *b*_5_ variants (45) is described in the Supporting Information. Briefly, *b*_5_ variants were expressed in *E. coli* JM109 cells, the cells were lysed by sonication, and the membrane pellet (which contains *b*_5_) was prepared. After solubilizing the membranes with sodium cholate, the homogenate was centrifuged (10^5^ × g), and the resulting supernatant was purified using DEAE anion exchange chromatography. UV-visible spectra of enzymes were recorded using either an OLIS Cary 14 or OLIS DW2a spectrophotometer (On-Line Instrument Systems). Concentrations of P450 17A1 were estimated from Fe^2+^-CO *versus* Fe^2+^ binding difference spectra using the excitation coefficient Δε_450-490_ = 91,000 M^-1^ cm^−1^ (66). The concentrations of *b*_5_ and its variants were calculated using the extinction coefficient ε_413_ = 117,000 M^-1^ cm^-1^ (67) or the difference extinction coefficient Δε_424-409_ = 180,000 M^-1^ cm^-1^ for the Fe^2+^ *versus* Fe^3+^ binding difference spectra (68). Apo-*b*_5_ was prepared from human WT *b*_5_ (holo-*b*_5_) by acid-acetone treatment according to a reported procedure (3, 41), and the concentration was estimated by a BCA assay using Pierce BCA Protein Assay Kit (Thermo Fisher Scientific), according to the manufacturer’s instructions.

### Preparation of Alexa 488-modified *b*_5_ variants

Labeling of WT *b*_5_ and apo-*b*_5_ was performed in a similar manner as previously reported (19). WT *b*_5_ (in 100 mM potassium phosphate buffer, pH 7.4) was mixed with Alexa Fluor 488 C5 maleimide (dissolved in dimethyl sulfoxide) at a 1 to 10 molar ratio, then incubated at room temperature overnight in amber glass. Unreacted dye was removed by passage through a Zeba Spin Desalting Column (7K MWCO, Thermo Fisher Scientific) according to the manufacturer’s instructions. For the modification of cysteine-containing *b*_5_ variants, proteins were treated with 10 mM DTT at room temperature for 30 min followed by the above desalting procedure. The reduced proteins were mixed with Alexa Fluor 488 C5 maleimide in a 1 to 5 molar ratio, incubated at room temperature for 2 h, and passed through desalting columns to remove the excess dye. Absorbance spectra of labeled proteins were measured using a Nanodrop spectrophotometer (Thermo Fisher Scientific), and the concentrations of Alexa 488 in the protein sample were calculated using ε_493_ = 72,000 M^-1^ cm^-1^. The purity of Alexa 488-modified *b*_5_ variants was analyzed by SDS-PAGE.

### Proteomic analysis

The in-gel digestion workflow was adapted from the method described by Shevchenko *et al*. (69) with minor changes. Coomassie brilliant blue-stained protein gel bands of interest were excised and diced into ∼1 mm^3^ cubes. The gel cubes were equilibrated for 5 min in 100 mM NH_4_HCO_3_ buffer (pH 8.0). Proteins were reduced in-gel with 4.5 mM DTT in 100 mM NH_4_HCO_3_ buffer (pH 8.0) at 55 °C for 20 min followed by alkylation with 10 mM iodoacetamide in 100 mM NH_4_HCO_3_ buffer (pH 8.0) at room temperature in the dark for 20 min. Gel pieces were destained with a 1:1 mixture (v/v) of 100% CH_3_CN and 50 mM NH_4_HCO_3_ (pH 8.0), dehydrated by the addition of 100% CH_3_CN, and dried in a centrifugal vacuum concentrator prior to proteolytic digestion. Trypsin Gold, mass spectrometry grade (Promega), diluted in 25 mM NH_4_HCO_3_ buffer (pH 8.0, 10 ng μl^−1^), was added to cover the dehydrated gel pieces, on ice. After 20 min, an additional 10 μl of 25 mM NH_4_HCO_3_ buffer (pH 8.0) was added to the gel pieces, and digestion was performed by incubation at 37 °C for 16 h. The resulting tryptic peptides were recovered by two extractions (15 min each) with 50 μl of 60% CH_3_CN/0.1% CF_3_CO_2_H (v/v). The extracts were combined and dried in a centrifugal vacuum concentrator. Peptides were reconstituted in 0.2% HCO_2_H (v/v) for analysis by liquid chromatography with tandem mass spectrometry. An analytical column was packed in-house with 20 cm of C_18_ reversed phase packing material (Jupiter, 3 μm beads, 300 Å, Phenomenex) directly into a laser-pulled emitter tip. Peptides were loaded on the capillary reversed phase analytical column (360 μm O.D. × 100 μm I.D.) using a Dionex Ultimate 3000 nanoLC and autosampler. The mobile phase solvents consisted of 0.1% HCO_2_H, 99.9% H_2_O (solvent A) and 0.1% HCO_2_H, and 99.9% CH_3_CN (solvent B) (all v/v). Peptides were gradient eluted at a flow rate of 350 nl min^-1^ using an 80 min gradient. The gradient consisted of the following mixtures: 1 to 64 min, 2 to 40% B; 64 to 71 min, 40 to 95% B; 71 to 72 min, 95-2% B; 72 to 80 min (column re-equilibration), 2% B (all v/v). Peptides were analyzed on an Orbitrap Exploris 480 mass spectrometer (Thermo Fisher Scientific), equipped with a nanoelectrospray ionization source. The data-dependent instrument method consisted of MS1 (R = 60000) using an AGC target of 3e6, followed by up to 15 MS/MS scans (R = 15,000) of the most abundant ions detected in the preceding MS scan. The intensity threshold for triggering data-dependent scans was set to 2.0e4, and the MS2 AGC target was 1e5. Dynamic exclusion was enabled with an exclusion duration of 10s, and higher-energy collisional dissociation collision energy was set to 28% nce.

For the identification of peptides, tandem mass spectra were searched with MSFragger (Fragpipe version 19.1, https://msfraggere.nesvilab.org) (70) using the MSFragger default configuration, including the following customizations. Data were searched against a *Homo sapiens* (human) subset database (downloaded 10 May 2021) from the UniprotKB protein database (www.uniprot.org). For analysis of mutant *b*_5_, the human database was appended with the T70C *b*_5_ sequence. Precursor mass tolerance was set to ±20 ppm and fragment mass tolerance to 20 ppm. Enzyme specificity was set to trypsin, and a maximum of two missed cleavages were allowed. Variable modifications included +15.9949 on Met (oxidation), +57.0214 on Cys (carbamidomethylation), +42.01056 on the N terminus (acetylation), +716.1095 on Cys and Lys (Alexa 488 fluorophore), +716.1095 on N-terminus (Alexa 488 fluorophore), +698.0989 on Cys and Lys (Alexa 488 fluorophore), +698.0989 on N terminus (Alexa 488 fluorophore). The target-decoy false discovery rate for peptide and protein identification was set to 1% for both peptides and proteins. Search results were assembled using Scaffold 5.1.2 (Proteome Software, https://www.proteomesoftware.com/products/scaffold-5). Peptide identifications were filtered following manual examination of assigned spectra.

### Protein complex modeling with AFM and Rosetta

**Figure 11:**
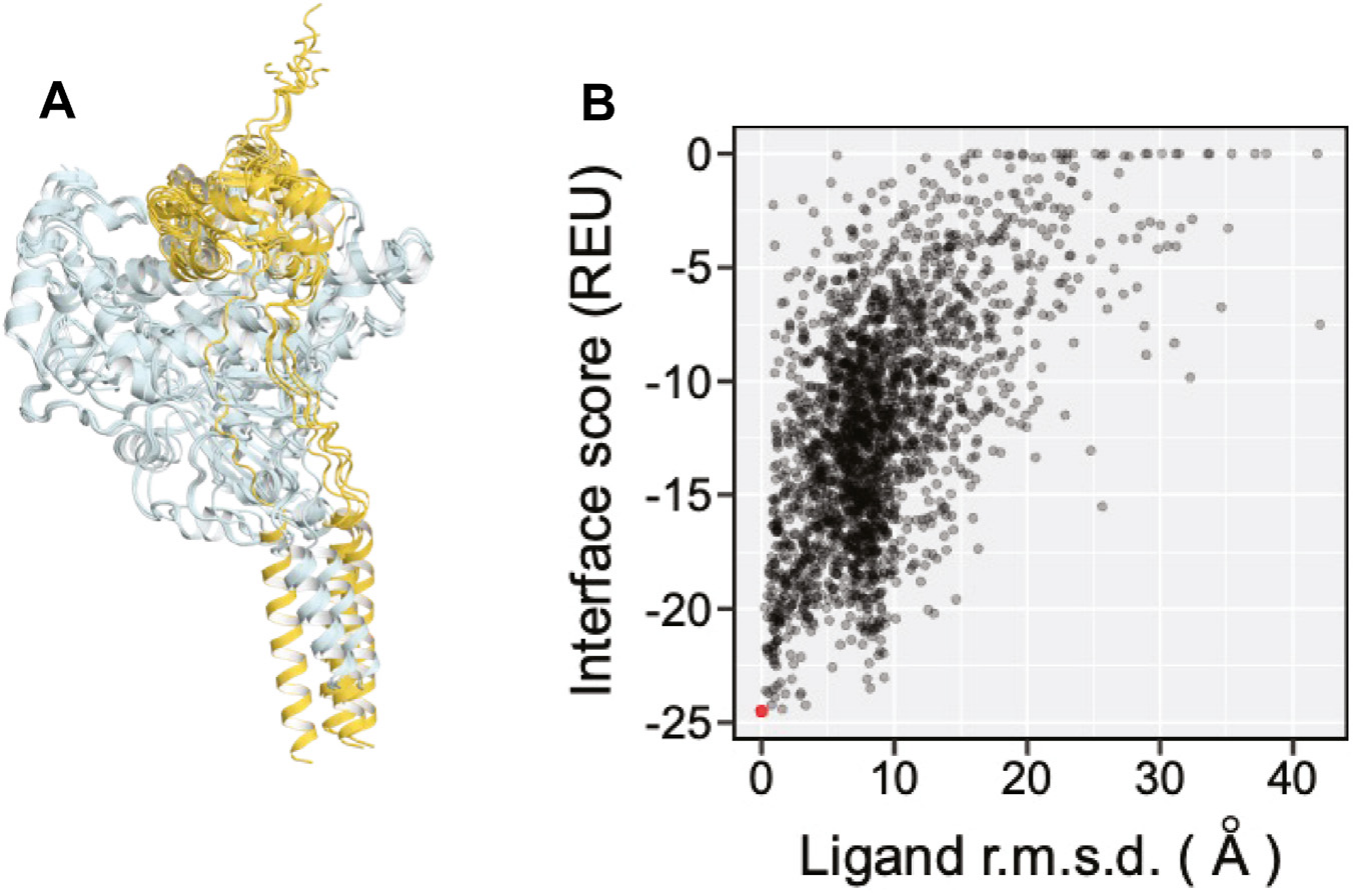
**Development of the AlphaFold-Multimer model.***A*, all five models of the *b*_5_-P450 17A1 complex as predicted by AlphaFold-Multimer (overlaid, *cyan*: P450 17A1, *yellow*: *b*_5_). The transmembrane helix of *b*_5_ in one of the models adopts a pose different from those in the other four models. *B*, scatter plot of Rosetta interface score *versus* ligand root-mean-square distance (r.m.s.d.). The *red dot* represents the docked complex with the lowest interface score (in Rosetta Energy Unit, or REU, more negative values indicate stronger binding). This complex was selected for structural analysis. Ligand r.m.s.d. measures the average deviation of ligand (*b*_5_) residues in all other docked models from the ligand residues in the lowest-energy model. The plot is indicative of a typical “energy funnel” where binding becomes stronger (more negative interface score) as r.m.s.d. becomes smaller, suggesting the convergence of docking.

A structural model of P450 17A1 bound with *b*_5_ was built using the AFM (37) protein structure prediction method, made available through the ColabFold interface (71) (https://colab.research.google.com/github/sokrypton/ColabFold/blob/main/AlphaFold2.ipynb). Because all five models generated by AFM are generally similar to each other (except that the transmembrane helix of *b*_5_ in one model adopts a different pose than the rest of the models, Fig. 11*A*), we selected the model top ranked by AFM for subsequent calculations. We grafted the heme prosthetic group for P450 17A1 from a crystal structure of P450 17A1 (Protein Data Bank ID: 3SWZ) (20) by aligning the crystal structure to the structure predicted by AFM (r.m.sd = 0.71 Å) using PyMOL. The heme prosthetic group for *b*_5_ was similarly grafted from the NMR structure of human *b*_5_ (Protein Data Bank ID: 2I96). The heme-grafted structural model for the *b*_5_-P450 17A1 complex was then subjected to relaxation into the Rosetta energy function (72). The goal of this energy-minimization step was to remove suboptimal geometries in the structure through minor adjustment of the coordinates of atoms causing the suboptimal geometries. Out of the 1000 models generated by the Rosetta FastRelax protocol (73), the lowest-scoring model was selected as the starting pose for further protein-protein docking using Rosetta (38, 74). Before docking, residues 1 to 46 in P450 17A1 and residues 1 to 8 and 95 to 134 were removed because they are either in the transmembrane helix and its associated loop or the N-terminal loop (residues 1–8 in *b*_5_) and we expect AFM to be less accurate in these regions. In protein-protein docking, we treated *b*_5_ as the ligand and set the values of perturbation flags as -dock_pert 3.8, -dock_mcm_trans_magnitude 0.7, and -dock_mcm_rot_magnitude 5.0. With protein-protein docking, we generated 2000 models of the *b*_5_-P450 17A1 complex with good convergence (Fig. 11*B*) and selected the model with the best interface score for structural analysis in this work. We note that after aligning the docked structure to the AFM-predicted structure based only on P450 17A1 atoms, the backbone r.m.sd for *b*_5_ is 1.1 Å. This result shows good agreement between deep-learning based prediction and biophysical modeling for *b*_5_-P450 17A1 structure.

### Titrations with fluorescent *b*_5_

P450 17A1 or hemin (0–180 nM final concentration) was titrated into a solution of Alexa 488-labeled WT *b*_5_ or each of the *b*_5_ variants (50 nM, based on the protein concentration) in 1 mM potassium phosphate buffer (pH 7.4) in a 1.0-cm quartz cuvette (Starna Cells, Inc, catalog # 29F-Q-10). Fluorescence spectra (excitation wavelength 493 nm, emission wavelength 500–600 nm) were recorded using an OLIS DM-45 spectrofluorimeter, with 1.24 mm slits (5.0 nm bandwidth). The data points were normalized to the initial fluorescence intensity at the emission maximum (508 nm). To examine the effect of nonlabeled *b*_5_, WT *b*_5_ (0–200 nM) was titrated into a complex of Alexa-modified *b*_5_ (50 nM) and P450 17A1 (150 nM) in 1 mM potassium phosphate buffer, pH 7.4 (Figs. S3*A*, and S6).

### 17α-OH pregnenolone lyase reaction catalytic assays

These reactions were performed as previously described, with some modifications (45). Briefly, 0.5 ml reconstituted systems were prepared in 50 mM potassium phosphate buffer (pH 7.4) with P450 17A1 (0.05 μM), 17α-OH pregnenolone (2.0 μM), Alexa 488-labeled or nonlabeled *b*_5_ (0.5 μM), POR (0.5 μM), and L-α-dilauroyl-*sn*-glycero-3-phosphocholine (30 μM). These reactions were preincubated at 37 °C for 5 min before initiation of the reactions by the addition of an NADPH-generating system (0.5 mM NADP^+^, 10 mM glucose 6-phosphate, and 2 μg ml^−1^ yeast glucose 6-phosphate dehydrogenase (75)). The reactions proceeded at 37 °C for 5 min before being quenched with 2 ml of CH_2_Cl_2_ and centrifuged at 10^3^g for 5 min. The product was derivatized with dansylhydrazine and analyzed by LC-MS according to a reported procedure (16, 45).

### Fluorescence polarization assays

Fluorescence polarization assays were performed in a 384-well, black, flat-bottom nonbinding plates (Greiner Bio One, Kremsmünster, Austria/Millipore-Sigma-Aldrich). P450 17A1 protein, P450 17A1 peptide, or WT *b*_5_ were mixed with Alexa 488-labeled proteins or peptides (10 nM) in 1 mM potassium phosphate buffer, pH 7.4 (final volume of 50 μl). After centrifugation at 10^3^g for 1 min, each plate was gently shaken for 5 min under the protection from light and read on a Synergy Neo plate reader (BioTek). Blank wells (without fluorescently labeled material) were included in each experiment and the parallel and perpendicular intensity (*I*_∥_ and *I*_⊥_, respectively) were subtracted from each data point. Polarization and anisotropy values were calculated using the following equations (76);

$$\text{Polarization}\;\left(\text{mP}\right)=\left(I_{∥}\;-\;I_{⊥}\right)\;/\;\left(I_{∥}+I_{⊥}\right)\times 10^{3}$$

$$\text{Anisotropy}\;\left(\text{mA}\right)=\left(I_{∥}\;-\;I_{⊥}\right)\;/\;\left(I_{∥}+2I_{⊥}\right)\times 10^{3}$$

The anisotropy data points were used for the analysis a quadratic equation in GraphPad Prism software (La Jolla, CA; https://www.graphpad.com/features) to calculate the *K*_d_ values using the following equation,

$$Y=A+\frac{\left(B-A\right)}{2E}\left[\left(Kd+E+X\right)-\sqrt{{\left(Kd+E+X\right)}^{2}-4EX}\right]$$

where *Y* is the observed anisotropy, *E* is the concentration of Alexa-488 modified *b*_5_ variants, *X* is the concentration of binding partner, *K*_d_ is the dissociation constant, and A and B are the anisotropies of free and bound species, set in Prism as: Y = A+(B-A)∗(0.5∗(1/E)∗((Kd+E+X)-sqrt((Kd+E+X)^∧^2-(4∗E∗X)))).

## Data availability

All data needed to evaluate the conclusions in the manuscript are present in the manuscript and/or the Supporting Information. The mass spectrometry proteomics data have been deposited to the ProteomeXchange Consortium *via* the PRIDE partner repository with the dataset identifier PXD047758.

## Supporting information

This article contains supporting information. Detailed experimental procedure for expression and purification of *b*_5_ and P450 17A1 and reduction of *b*_5_ by POR, figures of treatment of T70C *b*_5_ by reducing agents, absorbance spectra of P450 17A1 and hemin, additional flruoescence titrations, tandem mass spectra of modified Lys-containing peptides, SDS-PAGE of purified *b*_5_, reduction of POR-*b*_5_ complex, additional fluorescence polarizaiton assays, and tables of coveragge and list of peptides in proteomic analysis, primer sequences for mutagenesis, and rates of reduction of *b*_5_ by POR (36, 45, 46, 59).

## Conflict of interest

The authors declare that they have no conflict of interest with the contents of this article.
