# Supplementary material for: Proteomics, modeling, and fluorescence assays delineate cytochrome b5 residues involved in binding and stimulation of cytochrome P450 17A1 17,20-lyase
Source: J Biol Chem. 2024 Jan 26;300(3):105688. doi: 10.1016/j.jbc.2024.105688 (PMC10878793; doi:10.1016/j.jbc.2024.105688)
Supplement: Supporting information [file mmc1.docx]

**Supporting Information**

**Proteomics, modeling, and fluorescence assays delineate cytochrome *b*_5_ residues involved in binding and stimulation of cytochrome P450 17A1 17,20-lyase**

Yasuhiro Tateishi^1^, Stephany N. Webb^1^, Bian Li^2^, Lu Liu^1^, Kristie Lindsey Rose^1,3^, Micheal Leser^3^, Purvi Patel^3^, and F. Peter Guengerich^1^

**Table of Contents**

Experimental Procedures

Expression and purification of cytochrome *b*_5_ mutants in *Escherichia coli* S-3

Expression and purification of cytochrome P450 17A1 in *E. coli* S-4

Reduction of *b*_5_ by POR*………………………………………………………………………………* S-4

Supplementary Figures & Tables

Figure S1. Treatment of T70C *b*_5_ with reducing reagents. S-5

Figure S2. Absorbance spectra of P450 17A1 and hemin S-6

Figure S3. Fluorescence titration of Alexa 488-WT *b*_5_ with different conditions S-7

Figure S4. Fluorescence titration of Alexa 488 fluorophore with P450 17A1 or hemin S-8

Figure S5. Tandem mass spectra of modified Lys-containing peptides in Alexa 488-WT *b*_5_ S-9

Figure S6. Disruption of the binding Alexa 488-labeled *b*_5_ to P450 17A1 by unlabeled *b*_5_ S-11

Figure S7. SDS-PAGE of purified WT *b*_5_ and *b*_5_ variants. S-12

Figure S8. Reduction of POR–*b*_5_ complex S-12

Figure S9. Fluorescence polarization in stronger ionic strength buffer S-13

Figure S10. Binding of Alexa 488-K77C *b*_5_ to other P450 enzymes S-13

Figure S11. Alexa 488-modified P450 17A1 peptides did not bind to WT *b*_5_ S-14

Table S1. Coverage of Alexa 488 WT *b*_5_ and Alexa 488 T70C *b*_5_ in proteomic analysis S-15

Table S2. List of peptides detected from proteomic analysis S-16

Table S3. Sequences of primer pairs for generating Lys-to-Cys mutations of *b*_5_ S-17

Table S4. Rates of reduction of WT *b*_5_ and mutants by POR S-18

References used in Supporting Information S-18

Experimental Procedures

*Expression and purification of b*_5_ *mutants in Escherichia coli*

*E. coli* JM109 cells, transformed with the plasmids, were grown overnight in Luria Broth (LB) medium with 100 mg ml^-1^ ampicillin at 37 ℃, with shaking at 250 rpm. This overnight culture was seeded into 500 ml of Terrific Broth (TB) medium containing 2.0 g l^-1^ Bacto^TM^ peptone (BD), 0.4% glycerol (v/v), 100 µg ml^-1^ ampicillin, and 1.0 mM thiamine and grown at 37 °C with shaking at 250 rpm. Isopropyl β-D-1-thiogalactopyranoside (IPTG, 0.5 mM) and ẟ-aminolevulinic acid (ẟ-ALA, 0.5 mM) were added when the OD_600_ reached 0.5-0.6, and then the culture was incubated at 30 °C, 200 rpm for 42-48 h. The cells were harvested by centrifugation at 5,000 × g for 20 min at 4 °C and resuspended in 200 mM Tris HCl (pH 8.0) buffer containing 1.0 M sucrose and 1.0 mM EDTA, then lysed with chicken egg white lysozyme (12 µg ml^-1^) at 4 °C for 20-30 min. After centrifugation at 5,000 × g (4 °C, 20 min), the resulting pellet was resuspended in a sonication buffer (100 mM potassium phosphate, pH 7.4, 20% glycerol (v/v), and 6 mM Mg(OAc)_2_) containing 1 mM phenylmethylsulfonyl fluoride (PMSF) and protease inhibitor cocktail (Roche)) and sonicated. The homogenized pellet solution was centrifuged at 10^4^ × g at 4 ℃ for 20 min, and the supernatant was further centrifuged at 10^5^ × g at 4 ℃ for 2 h. The membrane pellet was re-solubilized in 20 mM potassium phosphate buffer (pH 7.4) containing 10% glycerol (v/v), 0.1 mM EDTA, and 0.5% sodium cholate (w/v) while stirring at 4 ℃ overnight. The homogenate was centrifuged at 10^5^ × g for 1 h, after which the supernatant was loaded onto an open bed 3 × 15 cm DEAE Sepharose Fast Flow column (Cytiva) previously equilibrated with four column volumes of 20 mM potassium phosphate buffer (pH 7.4) containing 10% glycerol (v/v), 0.1 mM EDTA, and 0.5% sodium cholate (w/v, buffer A). The column was washed with four column volumes of buffer A and then eluted utilizing a linear gradient of 0 to 100% buffer B (buffer A containing 0.5 M NaCl) over four column volumes. *b*_5_ variants (except for K39C *b*_5_) were further purified using an NGC™ Quest 10 chromatography system (Bio-Rad) equipped with a 5 ml Hi-Trap DEAE anion exchange column (Cytiva), eluting with a linear gradient 0 to 100% buffer B over 12 column volumes unless otherwise noted. Fractions were collected using a BioFrac™ fraction collector (Bio-Rad). K19C *b*_5_ was eluted using a gradient profile described as follows: 0 to 14% Buffer B over 10 column volumes, three column volumes of 14% Buffer B, 14 to 31% Buffer B over five column volumes, three column volumes of 31% Buffer B, and three column volumes of 100% Buffer B (all v/v). All purified *b*_5_ variants were dialyzed against 100 mM potassium phosphate buffer (pH 7.4).

*Expression and purification of cytochrome P450 17A1 in E. coli*

A codon-optimized pCW (Ori^+^) plasmid of P450 17A1 which has a (His)_6_ tag on the C-terminal was purchased from GenScript and expressed in *E. coli* JM109 cells, with purification exactly as described elsewhere (45).

*Reduction of b_5_ by POR*

The reduction of *b*_5_ by POR was examined in an OLIS RSM1000 stopped-flow spectrophotometer as described previously (46), mixing a pre-formed complex of *b*_5_ and POR (1:1 molar ratio) with 150 µM NADPH and collecting spectra (10^3^ s^-1^), which were analyzed using absorbance measurements at 409 and 424 nm and fitting to a single exponential equation. The assays were done in Ar-sparged buffer, rather than in anaerobic tonometers, in that the reoxidation rate of *b*_5_ is only ~0.1 s^-1^ in air (46,59) and an excess of NADPH was present.

Supplementary Figures & Tables

**Figure S1. Treatment of T70C *b*_5_ with reducing reagents.** T70C *b*_5_ (20 µl of 10 µM solution) was loaded before (lane 1) or after pretreatment with TCEP (1, 2, 10, and 20 mM, lanes 2 to 5) or DTT (1, 2, 10, and 20 mM, lanes 6 to 9) at room temperature for 30 min. The monomer (17 kDa) and dimer (34 kDa) bands are prominent.

**Figure S2. Absorbance spectra of P450 17A1 (A) and hemin (B).** Spectra were recorded in 1 mM potassium phosphate buffer (pH 7.4) using final concentrations of 0-0.18 µM. The wavelengths used for fluorescence titration assays are indicated in black (493 nm, for excitation) and red (508 nm, for emission) stipulated lines. The Δ absorbance of P450 17A1 at each wavelength is ≤ 0.016.

**Figure S3. Fluorescence titration of Alexa 488-WT *b*_5_ with different conditions.** *A* and *B*, Increasing amounts of WT *b*_5_ (0–0.2 µM) were added to a complex of Alexa 488-WT *b*_5_ (50 nM):P450 17A1 (150 nM) in 1 mM potassium phosphate buffer, and the increased fluorescence is indicated. *A*, emission spectra normalized to maximum fluorescence intensity (following the addition of 0.2 µM WT *b*_5_); *B*, normalized F/F_0_ plot at emission maximum wavelength (508 nm); *C* and *D*, Titrations were performed in 100 mM potassium phosphate buffer (pH 7.4) with Alexa 488-WT *b*_5_ (50 nM). *C*, emission spectra normalized to maximum fluorescence intensity; *D*, normalized F/F_0_ plot at emission maximum wavelength (508 nm) (red, ●). Titration data were also performed in 1 mM potassium phosphate buffer (black, ●, data is the same as shown in Fig. 3*C*) and included for comparison.

**Figure S4. Fluorescence titration of free Alexa 488 fluorophore (50 nM) with P450 17A1 (A) or hemin (B).** Titrations (in the absence of *b*_5_) were performed in 1 mM potassium phosphate buffer (pH 7.4).

**Figure S5. Tandem mass spectra of modified Lys-containing peptides in Alexa 488-WT *b*_5_.** The [M+3H]^3+^ (*A*–*E*) or [M+4H]^4+^ (*F*) precursor ions were selected for fragmentation, and the observed b- and y-type product ions are assigned to their corresponding *m/z* peaks in the mass spectrum. The amino acid sequences are provided above the annotated spectrum with the position of the Alexa 488 fluorophore (+698.0989) modification denoted by the asterisk (*), and sites of amide bond fragmentation are indicated with inter-residue brackets. The detected labeling positions are K10 (*A*), K19 (*B*), K24 (*C*), K33 (*D*), K39 (*E*), and K77 (*F*).

**Figure S6. Disruption of the binding Alexa 488-labeled *b*_5_ to P450 17A1 by unlabeled *b*_5_.** Increasing amounts of (non-labeled) WT *b*_5_ (0–0.2 µM) were added to a complex of Alexa 488-labeled *b*_5_ variants (50 nM):P450 17A1 (150 nM) in 1 mM potassium phosphate buffer, and the increased fluorescence is indicated. The emission spectra were normalized to maximum fluorescence intensity (following the addition of 0.2 µM WT *b*_5_).

**Figure S7. SDS-PAGE of purified WT *b*_5_ and *b*_5_ variants.** Proteins were pretreated with β-mercaptoethanol (10%, v/v) and separated on a 10% Bis-Tris gel with MES running buffer. The gel was imaged by total protein staining by SimplyBlue Safe Stain (ThermoFisher Scientific).

**Figure S8. Reduction of POR–*b*_5_ complex.** A mixture of POR and *b*_5_ (1 µM each) was mixed with an equal volume of 300 µM NADPH (both in 300 mM potassium phosphate buffer, pH 7.4). *A*, WT *b*_5_ (averaged data from 10 shots); *B*, K19C *b*_5_ (averaged data from 5 shots). Data were fit to a first-order process and rates were estimated to be 54 ± 2 s^-1^ (*A*) and 0.028 ± 0.001 s^-1^ (*B*), respectively.

**Figure S9. Fluorescence polarization in high ionic strength buffer.** Alexa 488-K77C *b*_5_ was incubated with P450 17A1 in 200 mM (*A*) or 1 mM (*B*) potassium phosphate buffer (pH 7.4). Note the difference in the *x*-axes. Panel B uses the same data as shown in Fig. 9*B*. The estimated *K*_d_ values are 445 nM (*A*) and 125 nM (*B*). Increasing the ionic strength of the buffer also decreased the dynamic range from Δ ~ 50 (*B*) to Δ ~30 (*A*).

**Figure S10. Binding of Alexa 488-K77C *b*_5_ to other P450 enzymes.** *A*, P450 3A4; *B*, P450 2C9; *C*, P450 2S1. The expression and purification of these P450s is described elsewhere (36). Estimated *K*_d_ values are shown in each panel.


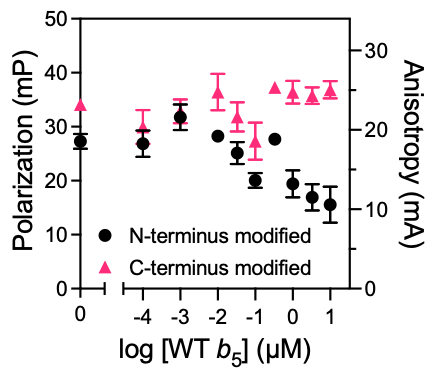


**Figure S11. Alexa 488-modified P450 17A1 peptides did not bind to WT *b*_5_.** Each point represents a mean ± SD of triplicate samples.

**Table S1. Coverage of Alexa 488 WT *b*_5_ and Alexa 488 T70C *b*_5_ in proteomic analysis.** Yellow shading indicates identified peptides. Green color indicates sites of modification (Alexa dye, oxidation (Met), carbamidomethylation/acetylation (Cys, N-terminal Ala).


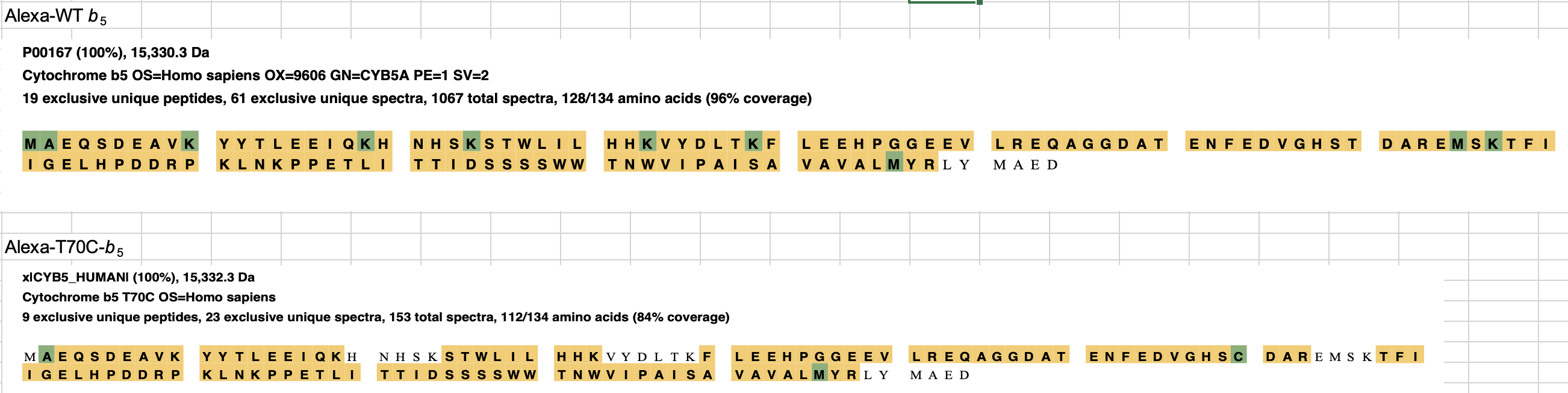


**Table S2. List of peptides detected from proteomic analysis.** Sites of modification are denoted with an asterisk (*).

| WT *b*_5_ |  |
| --- | --- |
| Peptide | Modification |
| (-)M*AEQSDEAVK(Y) | Oxidation |
| (M)*AEQSDEAVK(Y) | Acetyl |
| (M)AEQSDEAVK*(Y) | Alexa-488 |
| (M)AEQSDEAVK(Y) |  |
| (M)AEQSDEAVK*YYTLEEIQK(H) | Alexa-488 |
| (K)YYTLEEIQK(H) |  |
| (K)YYTLEEIQK*HNHSK(S) | Alexa-488 |
| (K)YYTLEEIQKHNHSK(S) |  |
| (K)HNHSK*STWLILHHK(V) | Alexa-488 |
| (K)HNHSKSTWLILHHKVYDLTK(F) |  |
| (K)STWLILHHK*(V) | Alexa-488 |
| (K)STWLILHHK(V) |  |
| (K)STWLILHHK*VYDLTK(F) | Alexa-488 |
| (K)STWLILHHKVYDLTK(F) |  |
| (K)STWLILHHKVYDLTKFLEEHPGGEEVLR(E) |  |
| (K)VYDLTK*FLEEHPGGEEVLR(E) | Alexa-488 |
| (K)VYDLTKFLEEHPGGEEVLR(E) |  |
| (K)FLEEHPGGEEVLR(E) |  |
| (K)FLEEHPGGEEVLREQAGGDATENFEDVGHSTDAR(E) |  |
| (R)EQAGGDATENFEDVGHSTDAR(E) |  |
| (R)EM*SK*TFIIGELHPDDRPK(L) | Oxidation, Alexa-488 |
| (R)EM*SKTFIIGELHPDDRPK(L) | Oxidation |
| (R)EMSKTFIIGELHPDDRPK(L) |  |
| (K)TFIIGELHPDDR(P) |  |
| (K)TFIIGELHPDDRPK(L) |  |
| (K)LNKPPETLITTIDSSSSWWTNWVIPAISAVAVALMYR(L) |  |
| (K)LNKPPETLITTIDSSSSWWTNWVIPAISAVAVALM*YR(L) | Oxidation |

Table S2 (continued)

| T70C |  |
| --- | --- |
| Peptide | Modification |
| (M)*AEQSDEAVK(Y) | Acetyl |
| (M)AEQSDEAVK(Y) |  |
| (M)AEQSDEAVKYYTLEEIQK(H) |  |
| (K)YYTLEEIQK(H) |  |
| (K)STWLILHHK(V) |  |
| (K)FLEEHPGGEEVLR(E) |  |
| (R)EQAGGDATENFEDVGHSC*DAR(E) | Alexa-488 |
| (R)EQAGGDATENFEDVGHSC*DAR(E) | Carbaminodimethyl |
| (R)EQAGGDATENFEDVGHSCDAR(E) |  |
| (K)TFIIGELHPDDR(P) |  |
| (K)TFIIGELHPDDRPK(L) |  |
| (K)TFIIGELHPDDRPK(L) |  |
| (K)LNKPPETLITTIDSSSSWWTNWVIPAISAVAVALM*YR(L) | Oxidation |
| (K)LNKPPETLITTIDSSSSWWTNWVIPAISAVAVALMYR(L) |  |

**Table S3. Sequences of primer pairs used for generating Lys-to-Cys mutations of *b*_5_.**

| Mutation | Forward (F) and Reverse (R) primers |
| --- | --- |
| K19C | CAC CCT AGA GGA GAT TCA GTG CCA CAA CCA CAG CAA GAG CA (F)  TGT TCT TGT TGT GGT TGT GGC ACT GAA TCT CCT CTA GGG TG (R) |
| K24C | CAG AAG CAC AAC CAC AGC TGC AGC ACC TGG CTG ATC CTG (F)  CAG GAT CAG CCA GGT GCT GCA GCT GTG GTT GTG CTT CTG (R) |
| K33C | AAA TTT GGT CAA ATC GTA CAC GCA GTG GTG CAG GAT CAG CCA GG (F)  CCT GGC TGA TCC TGC ACC ACT GCG TGT ACG ATT TGA CCA AAT TT (R) |
| K39C | CAC AAG GTG TAC GAT TTG ACC TGC TTT CTG GAA GAG CAT CCT GGT (F)  ACC AGG ATG CTC TTC CAG AAA GCA GGT CAA ATC GTA CAC CTT GTG (R) |
| K77C | ACA GAT GCC AGG GAA ATG TCC TGC ACA TTC ATC ATT GGG GAG CTC (F)  GAG CTC CCC AAT GAT GAA TGT GCA GGA CAT TTC CCT GGC ATC TGT (R) |

**Table S4. Rates of reduction of WT *b*_5_ and mutants by POR**

| *b*_5_/mutant | Rate, s^-1^ |
| --- | --- |
| WT | 54 ± 2 |
| K19C | 0.028 ± 0.001 |
| K24C | 3.1 ±0.1 |
| K33C | 0.027 ± 0.001 |
| K39C | 0.039 ± 0.001 |
| K70C | 0.016 ± 0.001 |
| K77C | 0.026 ± 0.001 |

References used in Supporting Infromation

36. Kim, D., Kim, V., Tateishi, Y., and Guengerich, F. P. (2021) Cytochrome *b*_5_ binds tightly to several human cytochrome P450 enzymes. *Drug Metab. Dispos.* **49**, 902-909

45. Guengerich, F. P., Tateishi, Y., McCarty, K. D., and Liu, L. (2023) Steroid 17α-hydroxylase/17,20-lyase (cytochrome P450 17A1). *Methods Enzymol.* **689**, 39-63

46. Guengerich, F. P. (2005) Reduction of cytochrome *b*_5_ by NADPH-cytochrome P450 reductase. *Arch. Biochem. Biophys.* **440**, 204-211

59. Yun, C.-H., Kim, K. H., Calcutt, M. W., and Guengerich, F. P. (2005) Kinetic analysis of oxidation of coumarins by human cytochrome P450 2A6. *J. Biol. Chem.*  **280**, 12279-12291
